# On a Source of Fallacy in Testing the Urine for Sugar

**Published:** 1847-06

**Authors:** G. Owen Rees

**Affiliations:** Assistant Physician to Guy’s Hospital, and Principal Medical Officer to the Pentonville Prison


*On a source of fallacy in testing the Urine for Sugar.* By G. Owen Rees, M.D., F R. S., Assistant Physician to Guy's Hospital, and Principal Medical Officer to the Pentonville Prison.—The many sources of fallacy connected with the examination of the urine are not, perhaps, so fully recognized by the profession as they ought to be.

These fallacies, inasmuch as they interfere with a due appreciation of symptoms, tend seriously to affect the correctness of diagnosis in urinary affections; and I therefore think it well to publish from time to time such facts as may come under my notice in relation to the subject.

A short time ago I received a specimen of urine said to contain both albumen and sugar in considerable quantity. The presence of sugar had been determined to the entire satisfaction of the medical attendant, who had demonstrated it to some friends, who had become equally well satisfied on the point. On examination, however, though I found albumen in abundance, I could not detect the slightest indication of sugar, notwithstanding that I used the same specimen of urine from which others had obtained satisfactory evidence.

On relating the result of my examination to my friend, from whose patient the urine had been obtained, I received an exact account of the manner in which sugar had been sought for, and was shewn the effect produced in the specimen by boiling with a solution of caustic potash—the excellent test proposed by Mr. Moore. I now perceived the whole contents of the tube to be of a deep brown colour, and should have been somewhat shaken in the fidelity of my own observation, had I not on a previous occasion applied this same test to the specimen, with a negative result.

I now observed that the liquor potassæ which had given the reaction had been kept in a white glass bottle, and immediately suspected that it might consequently contain lead; and that the dark colour produced, generally known as indicating the presence of sugar, might in this case be owing to the formation of sulphuret of lead; for lead dissolved in the liquor potassæ would unite to the sulphur of the albumen during the boiling, and I had satisfactorily ascertained the presence of albumen in this urine.

The liquor potassæ with which I had obtained a negative result was free from lead. On testing that of my friend, however, with hydro-sulphuret of ammonia, the black sulphuret of lead was thrown down in considerable quantity, shewing the correctness of the foregoing explanation.

It is very important that this source of fallacy should be borne in mind, as Mr. Moore's test is very much used for ascertaining the existence of diabetes mellitus: and liquor potassæ is unfortunately too often kept in white glass bottles, in tead of in those of green glass, which contain no lead in their composition on which the alkali can exercise a solvent action. The hydro-sulphuret of ammonia, applied as above described, will at once shew the practitioner whether or not his liquor potassæ be free from lead, in which case it produces no change of colour on being boiled with urine, even when albumen is present in large proportions.—*Lon. Med. Gaz.*

